# Antioxidant Properties of Pulp, Peel and Seeds of Phlegrean Mandarin (*Citrus reticulata* Blanco) at Different Stages of Fruit Ripening

**DOI:** 10.3390/antiox11020187

**Published:** 2022-01-19

**Authors:** Giulia Costanzo, Ermenegilda Vitale, Maria Rosaria Iesce, Daniele Naviglio, Angela Amoresano, Carolina Fontanarosa, Michele Spinelli, Martina Ciaravolo, Carmen Arena

**Affiliations:** 1 Department of Biology, University of Naples Federico II, Via Cinthia, 80126 Napoli, Italy; giulia.costanzo2@unina.it (G.C.); ermenegilda.vitale@unina.it (E.V.); 2 Department of Chemical Sciences, University of Naples Federico II, Via Cinthia, 80126 Napoli, Italy; iesce@unina.it (M.R.I.); daniele.naviglio@unina.it (D.N.); angela.amoresano@unina.it (A.A.); carolina.fontanarosa@unina.it (C.F.); mic.spinelli87@gmail.com (M.S.); martinaciaravolo@gmail.com (M.C.)

**Keywords:** ascorbic acid, antioxidant activity, chlorophylls, carotenoids, phenolic compounds, flavonoids, condensed tannins, Phlegrean mandarin

## Abstract

In this work, we assess the potential of waste products of Phlegrean mandarin (*Citrus reticulata* Blanco), namely seeds and peel, to be reutilized as a source of bioactive compounds beneficial for the human diet. Starting from the evidence that the by-products of this specific cultivar are the most powerful sources of antioxidants compared to pulp, we have investigated if and how the bioactive compounds in peel and seeds may be affected by fruit ripening. Three stages of fruit ripening have been considered in our study: unripe fruits = UF, semi-ripe fruits = SRF, ripe fruits = RF. The overall results indicated that RF showed the highest concentration of antioxidants. Among fruit components, peel was the richest in total antioxidant capacity, total polyphenol content, total flavonoids, total chlorophylls and carotenoids, while seeds exhibited the highest concentration of total condensed tannins and ascorbic acid. The liquid chromatography-tandem mass spectrometry (LC-MS/MS) assay indicates the occurrence, in peel extracts, of 28 phenolic compounds, mainly flavonoids (FLs); in seeds, 34 derivatives were present in the first stage (UF), which diminish to 24 during the ripening process. Our data indicated that the content of phytochemicals in citrus strongly varies among the fruit components and depends on the ripening stage. The higher antioxidant activity of peel and seeds, especially in RF, encourage a potential use of by-products of this specific citrus cultivar for industrial or pharmacological applications. However, to maximize the occurrence of desired bioactive compounds, it is important also to consider the ripening stage at which fruits must be collected.

## 1. Introduction

Vegetables and fruits are important sources of nutrients, dietary fiber, and phytochemicals, which promote several benefits for human health [1], mainly due to their ability to remove the reactive oxygen species (ROS) associated to the development of chronic disease [2,3].

Indeed, an increasing consumption of foods rich in antioxidants reduces the risk of several chronic-degenerative diseases considered the most prevalent causes of death in the world [4] and counteracts the cellular aging [1]. Moreover, antioxidants and other phytochemicals, when added to food, delay rancidity and the formation of oxidation products, preserving food quality and increasing their shelf life [5].

In the human diet, the assumption of citrus fruits represents one of the most important sources of antioxidants, mainly vitamin C [6], which is one of the most diffuse natural antioxidants.

Citrus represents one of the world’s major horticultural crops, with a global production over 100 million metric tons per year [7]. The fruits contain several kinds of phytochemicals which enrich the human diet resulting in a potential improvement of health [4,8,9].

Previous studies focused on several citrus species showed that the edible portion (pulp or juices) was rich in phenolic compounds, mainly phenolic acids and flavanones [10,11,12,13,14].

Generally, citrus pulp is consumed directly or as juice; for this reason, many citrus parts, such as peels and seeds are discarded and considered wastes [15]. Given the high quantity of product utilized every year, the citrus waste disposal represents a serious environmental problem because the amount of food wastes varies from 8 to 20 million tons per year globally [16]. This encourages the adoption of recycling strategies to promote potential innovative uses of citrus by-products [17,18]. Recent studies on citrus bioactive compounds have reported that peels and seeds are very interesting fruit components, potentially useful in a wide field of application [7,14,19,20,21,22]. However, up to now, limited studies have analyzed and compared the phytochemical content in peel, pulp, and seeds in the vision of a sustainable use of citrus wastes [22]. Moreover, some cultivars, such as the Phlegrean mandarin (*Citrus reticulata* Blanco), are particularly rich in secondary compounds, contained especially in seeds, which may be considered as a source of antioxidants, reporting the attention on citrus waste products [23].

The Phlegrean mandarin is a typical cultivar of the Phlegrean area (Naples, Southern Italy). The volcanic nature of the soil, joined with the mild climate of this area, gives peculiar properties to the fruits of this cultivar. Based on this, deep investigations on the potentiality of Phlegrean mandarin have become attractive, particularly to explore if and how the antioxidant activity may change during the ripening since this specific cultivar represents an unexploited resource typical of the Campania region (Southern Italy). Fruit ripening modifies several biochemical, physiological, and structural properties and these may affect fruit quality [24]. During ripening, mandarins exhibit a color scale ranging from green to deep orange or reddish, depending on the concentration of chlorophylls, carotenoids, and anthocyanins. The increase of carotenoids during ripening is also associated to changes of other bioactive compounds (i.e., polyphenols, flavonoids, and ascorbic acid), and to qualitative fruit traits, such as astringency and acidity, which decrease with ripening [25,26]. Besides ripening, climate, soil type, cultivation practices and cultivar also affect the final fruit characteristics [7].

This work evaluates the composition of phytochemicals, mainly antioxidants, in the peel, pulp, and seed of Phlegrean mandarin and assesses how the different fruit ripening stage affects the bioactive compound synthesis with the specific aim to identify the stage (balsamic period) and the fruit component most abundant with compounds beneficial for the human diet.

An additional aspect related to this research is the re-evaluation of mandarin by-products. In food manufacturing, citrus is mainly used to produce fresh juice or citrus-based drinks, with many wastes such as peels, pulp residuals and seeds accumulated annually. According to previous findings, our study proposes that these parts could be used as a natural source of antioxidants [7,14,20], thus encouraging recycling policies and reducing the environmental impact due to waste disposal [15,27]. Currently, citrus wastes are used in biogas production [17,27], ruminant feeding [18], and essential oil extraction [28]. Therefore, the perspective of utilizing the citrus wastes as a source of phytochemicals as a supplement for the human diet represents an attractive new application [29,30,31].

Furthermore, our study demonstrates that the pharmacological potentiality of mandarin by-products, peel and seeds, varies with fruit ripening, providing the evidence that maturation of fruit is an important parameter to consider when utilizing mandarin waste components as resources for phytochemicals or additives in producing functional food for human and animal feeding.

## 2. Materials and Methods

### 2.1. Plant Material and Sample Preparation

Fruits of *Citrus reticulata* Blanco used in our study originate from Naples in Southern Italy, in the peculiar area of the Phlegrean fields (latitude 40°49′39.65″; longitude 14°08′20.55″). The Phlegrean mandarin is a typical cultivar of the Phlegrean volcanic area (Naples, Southern Italy), characterized by a mild climate and very fertile volcanic soils [23].

Citrus fruits used for the analyses were collected during the seasons 2019–2020, in October and December, from three different trees (three fruits for each tree) at three different ripening stages: unripe fruit (UF), semi-ripe fruit (SRF) and ripe fruit (RF). To reduce the differences within the mandarin plantation, we selected the trees for the sampling based on the same age and exposure to sunlight, which represent a crucial factor for fruit ripening. In addition, we chose the trees in a family-run private company, and the fruits used for the experiment came from trees not treated with pesticides.

The samples were placed in plastic bags filled with dry ice, quickly transferred to the laboratory, and stored at −80 °C for subsequent analysis. In the laboratory, fruits of each ripening stage were washed with distilled water, separated into the peel, pulp and seeds, and grounded in liquid nitrogen using a mortar and pestle. The powder of each fruit component at different ripening stages was placed in test tubes and stored at −20 °C until analysis. For each ripening stage, a total of eight laboratory replicates, obtained by mixing three biologically distinct samples (three fruits) for each fruit part, were analyzed. We have analyzed fruits collected in both seasons, 2019 and 2020. As we did not find significant differences in the investigated parameters, we pooled the mandarin cohorts collected in different seasons.

### 2.2. Photosynthetic Pigments Content Analysis

The determination of total chlorophylls and carotenoids contained in peel, pulp and seeds was performed according to Lichtenthaler [32]. Briefly, pigments were extracted from about 0.010 g of powdered sample in ice-cold 100% acetone for 5 min and then centrifuged (Labofuge GL, Heraeus Sepatech, Hanau, Germany) at 5000 rpm for 5 min. The absorbance was measured by a spectrophotometer (Cary 100 UV-VIS, Agilent Technologies, Santa Clara, CA, USA) at wavelengths of 470, 645 and 662 nm, and pigment concentration was expressed as mg g^−1^ fresh weight (FW).

### 2.3. Phenolic Extraction

Peel, pulp and seeds were finely ground with liquid nitrogen, using a mortar and pestle. Then, 0.20 g of this material was extracted in 2 mL of aqueous 80% methanol. The extracts were kept for 1 h at 4 °C and then centrifuged at 11.000 rpm for 5 min. They were stored at 4 °C until analyzed and utilized to quantify total polyphenols, flavonoids content and total condensed tannins.

### 2.4. Determination of Total Polyphenols

Total polyphenol content was measured following the procedure reported in Costanzo et al. [23]. An aliquot (274 μL) of a suitable diluted methanol sample was added to 274 μL of the Folin–Ciocalteu reagent. The mixture was shaken and allowed to stand for 3 min, before adding 1.452 mL of 700 mM sodium carbonate (Na_2_CO_3_) solution. Samples were incubated for 2 h in darkness. Then, the absorbance was measured at 765 nm by a spectrophotometer (UV-VIS Cary 100, Agilent Technologies, Palo Alto, CA, USA). The total polyphenol content was calculated using gallic acid standard curve and expressed as mg Gallic Acid Equivalents (GAE) g^−1^ FW.

### 2.5. Flavonoid Content and Total Condensed Tannins

Total flavonoid content was measured according to Moulehi et al. [7] and Sun et al. [33]. Briefly, 250 µL of a diluted methanol sample was mixed with 75 µL of 5% NaNO_2_ (sodium nitrite). After 6 min, 150 µL of 10% AlCl_3_ (aluminium chloride) and 500 µL NaOH (1 M) were added to the mixture. Lastly, the mixture was adjusted with distilled water to a final volume of 1.525 mL. The absorbance was read at 510 nm. Total flavonoid content was calculated using a catechin standard curve and expressed as mg catechin equivalents per gram of fresh weight (mg CE g^−1^ FW).

Total condensed tannins were evaluated using the modified vanillin assay reported in Sun et al. [33]. Then, 1.25 mL of 1% methanol vanillin solution and concentrated H_2_SO_4_ (1:1 *v*/*v*) were added to 500 µL of powdered sample, diluted with aqueous 80% methanol. After 15 min, the absorbance was measured at 500 nm against methanol as a blank. Total condensed tannins were expressed as mg catechin equivalents per gram of fresh weight (mg CE g^−1^ FW) through the calibration curve with catechin.

### 2.6. Ascorbic Acid Content

The ascorbic acid (AsA) content was evaluated using the Ascorbic Acid Assay Kit (MAK074, Sigma-Aldrich, St. Louis, MO, USA) according to methods reported in Arena et al. [34].

The assay reaction was performed by adding the kit reagents to the samples. More specifically, 10 mg of each sample were mixed in four volumes of cold AsA buffer and centrifuged at 13.000 rpm for 10 min at 4 °C to eliminate any traces of insoluble material. The surnatant was mixed with AsA assay buffer up to a final volume of 120 μL. In this assay, the AsA concentration was determined by a coupled enzyme reaction, which develops a colorimetric (570 nm) product, corresponding to the amount of ascorbic acid contained in the sample (0.01 g). The concentration of ascorbic acid in each sample was referred to a standard curve and expressed in ng μL^−1^.

### 2.7. Antioxidant Activity Assays

In order to obtain more robust data, the antioxidant activity was tested with Ferric Reducing Antioxidant Power (FRAP) assay and 1,1-diphenyl-2-picrylhydrazyl (DPPH) assay.

#### 2.7.1. FRAP Assay

The antioxidant activity was evaluated by the Ferric Reducing Antioxidant Power (FRAP) assay, as described in George et al. [35]. Briefly, 0.25 g of powdered sample was mixed with 5 mL of 60:40 (*v*/*v*) methanol/water solution. The extracts were kept for 1 h on ice and then centrifuged at 14.000 rpm for 15 min, at 4 °C. An aliquot (150 μL) of extract was mixed with the FRAP reagents (2.5 mL of 300 mM acetate buffer pH 3.6, 250 μL of 10 mM tripyridyltriazine (TPTZ) and 250 μL of 12 mM FeCl_3_) and incubated for 1 h in the dark. Finally, the absorbance was measured at 593 nm by a spectrophotometer (UV-VIS Cary 100, Agilent Technologies, Palo Alto, CA, USA). The antioxidant capacity was calculated using a Trolox standard curve and expressed as μmol Trolox equivalents (μmol TE g^−1^ FW).

#### 2.7.2. DPPH Assay

Free radical scavenging activity of peel, pulp and seeds extracts was evaluated using the 1,1-diphenyl-2-picrylhydrazyl (DPPH) assay, according to Dudonné et al. [36]. An aliquot (67 μL) of a suitable diluted methanol sample was added to 2 mL of 6 × 10^−5^ M DPPH methanolic solution. The mixture was shaken vigorously and incubated at 37 °C for 20 min. The absorbance of the resulting solution was measured at 515 nm and converted into the percentage of inhibition of DPPH radicals using the following equation:

Inhibition (%) = [(Ab_blank_ − Ab_sample_)/Ab_blank_] × 100,

where Ab_blank_ is the absorbance of the blank and Ab_sample_ is the absorbance of the tested methanolic extract. Trolox was used as the positive control.

### 2.8. Characterization of Phenolic Compounds in Peel and Seeds by Liquid Chromatography Tandem Mass Spectrometry (LC-MS/MS) Analysis

All standards were purchased from Sigma-Aldrich (St. Louis, MO, USA). All the solutions and solvents were of the highest available purity and were suitable for LC–MS analysis and purchased from J. T. Baker (Phillipsburg, NJ, USA).

#### 2.8.1. Preparation of Standard Solutions and Samples

The stock solutions were prepared by adding 1.00 mL aliquots of each analyte to a 10 mL volumetric flask and bringing the standard to volume with methanol to yield a standard solution with 1000 µg L^−1^ of each analyte. The stock solutions were stored at −20 °C until the analysis. Quantitative analysis was performed by construction of calibration curves for a set of standard molecules selected for the different class of analytes under investigation. Standard mixtures were prepared by series dilution as follows: 2.5, 5.0, 25, 50, 250, 500 µg L^−1^.

Extracts in methanol of peel and seeds, prepared according to the procedure in Section 2.3, were further diluted 1:10 in the same solvent and filtered and centrifuged at 10.000 rpm for 10 min. The supernatant was then directly transferred into HPLC auto sampler and 1 µL of supernatant was analyzed by liquid chromatography-tandem mass spectrometry (LC-MS/MS) assay.

#### 2.8.2. LC-MS/MS Instrumentation and Conditions

One microliter of supernatant was analyzed by using an AB-sciex 5500 QTRAP^®^ system with a HPLC chromatography system Exion LC™. The mobile phase was generated by mixing eluent A (0.1% formic acid in water) and eluent B (0.1% formic acid in acetonitrile) and the flow rate was 0.200 mL min^−1^. Chromatographic gradient was from 20% to 90% B in 4 min, held for 2 min, then returned to 20% B in 1 min. Tandem mass spectrometry was performed using a Turbo V^TM^ ion source operated in positive ion mode, and the multiple reaction monitoring (MRM) mode was used for the selected analytes. Supplementary Table S1 provides a list of precursor ions, product ions, collision energy and declustering potential parameters.

The extracted mass chromatogram peaks of metabolites were integrated using Skyline software for data processing.

#### 2.8.3. Quantification of Analytes

The first step for the setting of mass spectral analysis consisted of MRM detection of the analytes individually injected to establish the optimal instrument settings for each compound. Standard calibration curves for the selected set of molecules were constructed by plotting peak areas against concentration (µg L^−1^), and linear functions were applied to the calibration curves. The coefficients of determination (R2) were greater than 0.99 for all analytes.

### 2.9. Statistical Analysis

Statistical analysis was performed using Sigma Plot 12.0 (Jandel Scientific, San Rafael, CA, USA). The effect of the different independent factors, namely fruit component (C) and ripening stages (RS), as well as their interaction, on the bioactive compounds content was assessed by two-way ANOVA followed by Duncan’s multiple comparison test, for all pairwise multiple comparison procedures, based on a significance level of *p* < 0.05. Whenever the interaction between C and RS was significant, data were subjected to one-way ANOVA and multiple comparison tests were performed with the Duncan’s coefficient. The normal distribution of data was verified by Shapiro–Wilk and Kolmogorov–Smirnov tests. All data were expressed as means ± standard error (SE) (*n* = 8).

The visualization of overall parameters was obtained by a heatmap function. The heatmap was plotted by using the ClustVis program package (https://biit.cs.ut.ee/clustvis/online, accessed on 20 December 2021) and clustering both rows and columns with correlation distance and complete linkage. The sequential palette evidences the numeric differences of the data matrix: blue and red colors indicate lower and higher values respectively.

## 3. Results

### 3.1. Total Phenolic Compounds

**Figure 1:**
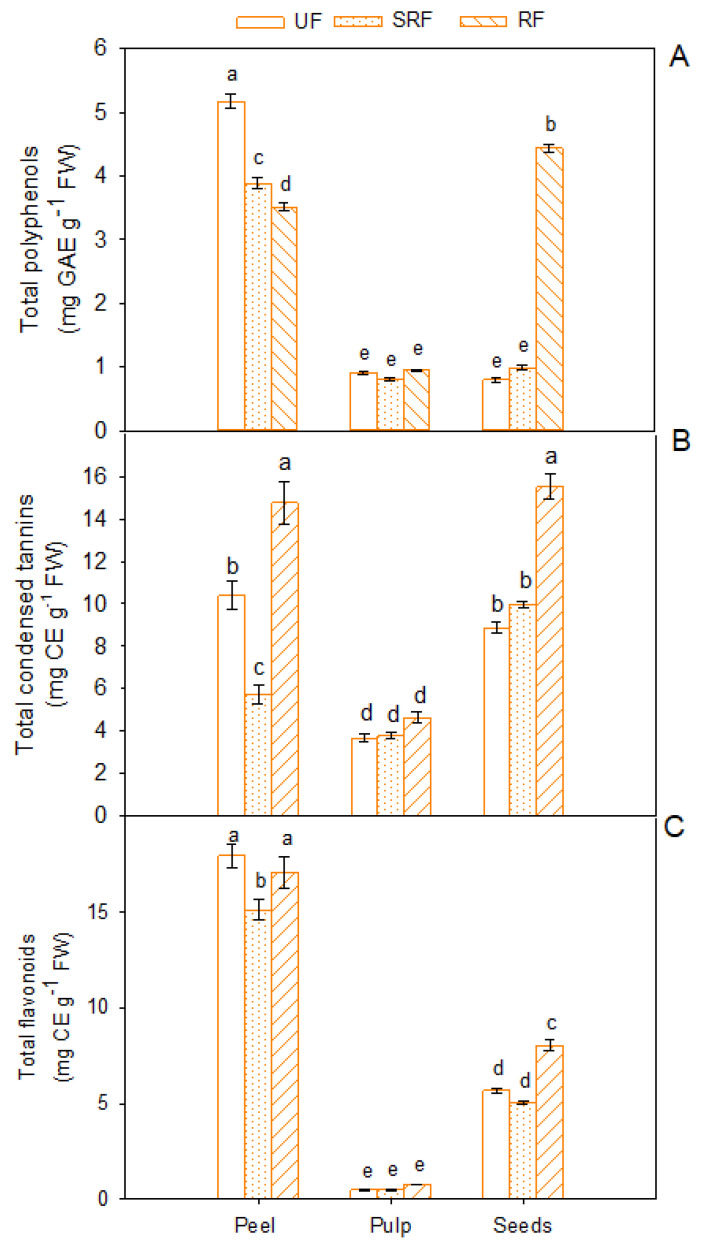
Total polyphenols (**A**), total condensed tannins (**B**) and total flavonoids (**C**) in peel, pulp, and seeds of unripe (UF), semi-ripe (SRF) and ripe fruits (RF), of Phlegrean mandarin. Each bar represents the mean ± SE (*n* = 8). Different letters indicate significant differences (*p* < 0.05), according to Duncan’s post-hoc test. GAE: gallic acid equivalents; CE: catechin equivalents; FW: fresh weight.

Total polyphenol content strongly varied depending on fruit component and ripening stage (Figure 1A).

The highest amount (*p* < 0.05) of total polyphenols was found in peel for unripe (5.17 ± 0.11 mg GAE g^−1^ FW), semi-ripe (3.89 ± 0.08 mg GAE g^−1^ FW) and mature fruits (3.51 ± 0.06 mg GAE g^−1^ FW). Conversely to pulp, where no statistically significant difference was detected, ripeness significantly affected the total polyphenol content in seeds. Indeed, seeds at the mature stage exhibited a surprisingly high total phenol content (4.44 ± 0.06 mg GAE g^−1^ FW) compared to that found in UF and SRF.

Condensed tannin content varies significantly among different fruit components, showing elevated concentrations in peel and seeds compared to pulp. In addition, significant differences were also evidenced with fruit ripening. In particular, tannin concentration was high in peel (14.7 ± 0.70 mg CE g^−1^ FW) and seeds (15.5 ± 0.58 mg CE g^−1^ FW) compared to pulp in mature fruits (Figure 1B).

Among the different fruit parts, peel exhibited the highest total flavonoid content (*p* < 0.01), followed by seeds. However, while in peel and pulp there is no relationship between flavonoid content and ripening, in seeds the highest polyphenol content was observed in mature fruits (Figure 1C).

### 3.2. Ascorbic Acid

**Figure 2:**
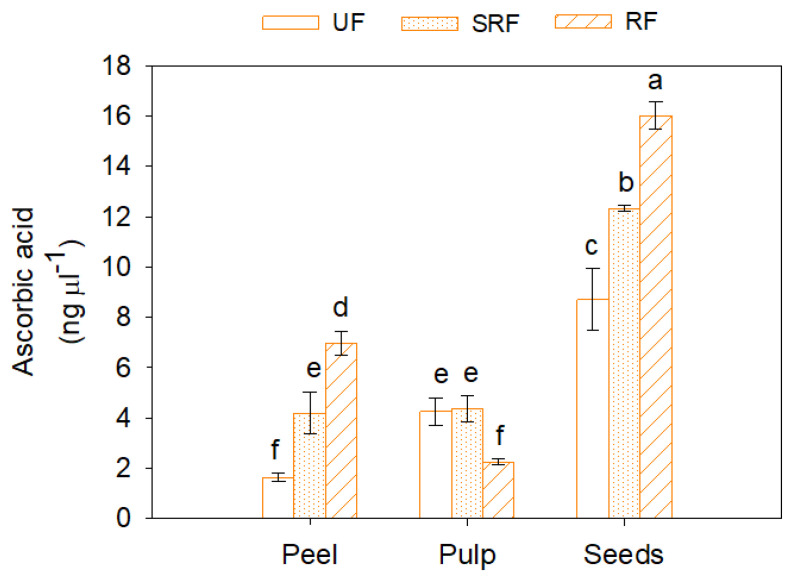
Ascorbic acid content in peel, pulp, and seeds of unripe (UF), semi-ripe (SRF) and ripe fruits (RF), of Phlegrean mandarin. Each bar represents the mean ± SE (*n* = 8). Different letters indicate significant differences (*p* < 0.05), according to Duncan’s post-hoc test. FW: fresh weight.

Ascorbic acid (AsA) content strongly varied (*p* < 0.05) among different fruit ripening stages and different fruit parts (Figure 2). Moreover, in this case, a very high content of ascorbic acid was found in peel and seeds, with a progressive increase as the ripening progressed. In peel and seeds of mature fruits, the highest AsA concentration was found (7.0 ± 0.2 ng µL^−1^ and 16.0 ± 0.55 ng µL^−1^, respectively). On the other hand, in pulp, an opposite trend was observed with lower AsA values as ripening progressed.

### 3.3. Sample Antioxidant Activity

**Figure 3:**
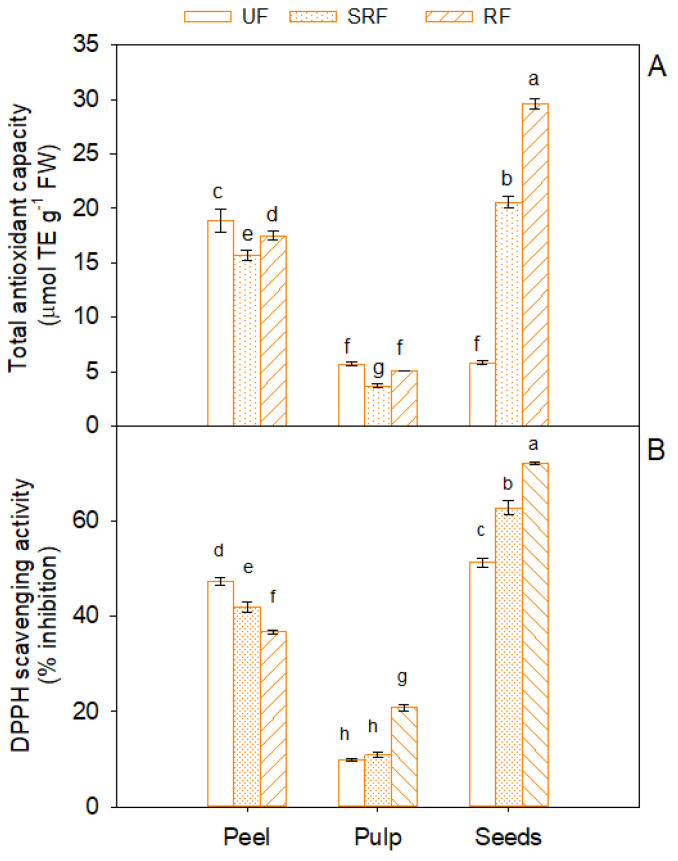
Total soluble antioxidant capacity (**A**) and DPPH (1,1-diphenyl-2-picrylhydrazyl) scavenging activity (**B**) in peel, pulp, and seeds of unripe (UF), semi-ripe (SRF) and ripe fruits (RF), of Phlegrean mandarin. Each bar represents the mean ± SE (*n* = 8). Different letters indicate significant differences (*p* < 0.05), according to Duncan’s post-hoc test. TE: Trolox equivalents; FW: fresh weight.

Total soluble antioxidant capacity determined by FRAP assay (Figure 3A) significantly varied with ripeness. Similarly to total polyphenols, in all ripening stages it was higher in the peel and seeds compared to pulp, reaching the highest value in seeds of ripe fruits (29.6 ± 0.56 μmol Trolox eq g^−1^ FW). It is interesting to observe that the total antioxidant capacity significantly increased in seeds with ripening (*p* < 0.05), conversely to peel, where the highest (*p* < 0.05) antioxidant capacity was detected in unripe fruits. The DPPH assay also confirmed that scavenging activity of free radicals changed with ripeness and showed an opposite trend in peel and fruits (Figure 3B). More specifically, with fruit ripening, while it decreased in peel, it increased in seeds reaching the highest values at full fruit maturity (72% of inhibition at RF stage).

### 3.4. Total Chlorophyll and Carotenoid Content

**Figure 4:**
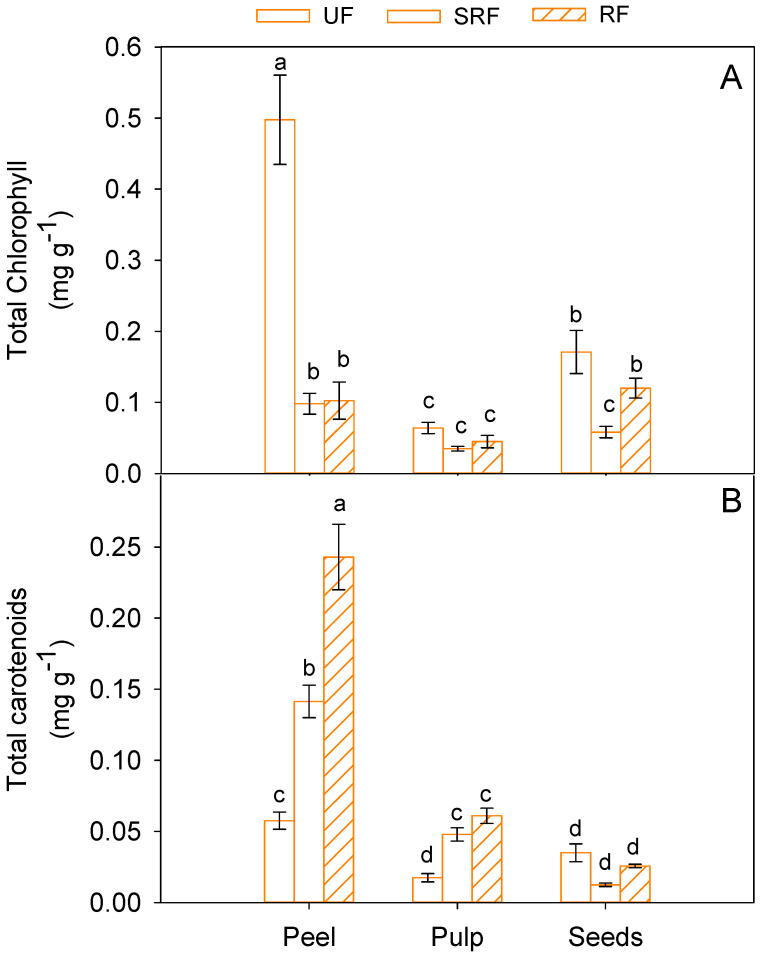
Total chlorophyll (**A**) and carotenoids (**B**) in peel, pulp, and seeds of unripe (UF), semi-ripe (SRF) and ripe fruits (RF), of Phlegrean mandarin. Each bar represents the mean ± SE (*n* = 8). Different letters indicate significant differences (*p* < 0.05), according to Duncan’s post-hoc test. GAE: gallic acid equivalents; CE: catechin equivalents; FW: fresh weight.

Total chlorophyll and carotenoid content varied significantly (*p* < 0.05) in the different fruit components and along the ripening stage. In unripe fruits, peel was the richest in chlorophylls (0.50 ± 0.06 mg g^−1^ FW), and its concentration strongly declined in semi-ripe (SMF) and ripe fruits (RF) (Figure 4A). The carotenoids also showed a higher amount in peel than in other fruit components, but they exhibited an opposite trend compared to chlorophylls, with the highest values in ripe fruits (0.24 ± 0.02 mg g^−1^ FW) (Figure 4B). In pulp, the chlorophyll content showed no relationship with the ripening stage, while the carotenoid amount increased significantly (*p* < 0.05) as ripening progressed.

### 3.5. Characterization of Phenolic Compounds in Peel and Seeds at Diverse Ripening Stages

Qualitative analysis and identification of the phenolic compounds contained in peel and seeds were carried out by using LC-MS/MS in both positive (ESI+) and negative (ESI) ionization modes.

A set of targeted molecules were explored by mass spectrometry in multiple reaction monitoring as reported in the supplementary Table S1, taking advantage of high performances of triple quadrupole MS. For each molecule, specific transition precursor ions/fragment ions were selected (Supplementary data, Table S1). Quantitative analyses were performed by using the external standard method. The polyphenol composition of peel and seeds during the three ripening stages has been reported in Table 1 and Table 2, respectively.

In the current study, a total of 28 phenolic compounds were identified in peel (Table 1). In the peel extracts, the main products were flavonoids with multiple substitution patterns including anthocyanins, flavanones, flavanols, flavonols and one flavone (polymethoxylated flavone sinensetin). They were generally glycosylated with glucose or ramnose or arabinose, and in some cases also diglycosylated (rutin). Two phenolic acids (one accompanied by the corresponding aldehyde), one dihydrochalcone (phloretin) and its glycosylated form (phloredzin) and one cinnamic acid (chlorogenic acid) were also found. As regards polyphenols, analysis evidenced the presence of catechin-3-gallate (CG) and epicatechin-3-gallate (ECG) and the tannin valoneic acid dilactone.

The peel phenolic composition was strongly affected by fruit ripening stage. The most interesting stage seems to be the SRF for some compounds, namely delphinidin-3-*O*-glucoside, cyanidin-3-*O*-glucoside, delphinidin rutinoside, naringin, quercetin-3-glucoside, rutin, that reach the highest concentration.

As shown in Table 2, the composition of phenolic extracts from seeds presents 34 derivatives in the first stage (UF) that reduce to 24 during the ripening process. In addition to those found in the peels, more polyphenols (catechin-3-gallate, epicathechin (EC)-, epigallocatechin (EGC)- and gallocatechin (GC) 3-gallate) and one isoflavone derivative (6-malonyldaidzin) were also detected.

### 3.6. Heatmap Analysis

**Figure 5:**
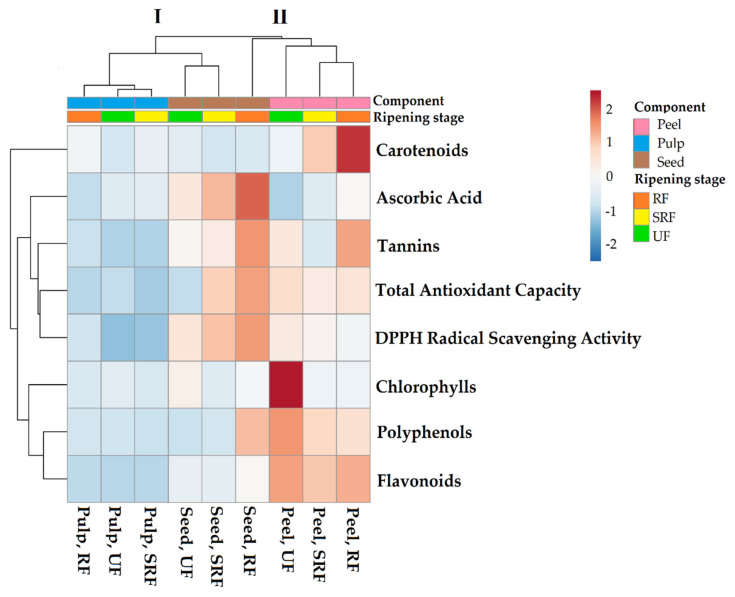
Heatmap analysis of bioactive compounds in different fruit components (peel, pulp, and seed) of Phlegrean mandarin at different ripening stages (UF—unripe fruit, SRF—semi-ripe fruit, and RF—ripe fruits). Numeric differences within the data matrix are shown by the sequential color palette: blue and red indicate decreasing and increasing values, respectively. Sample groups are clustered in the columns by the two independent factors, Component and Ripening Stage.

Figure 5 shows an overview of the bioactive compounds of Phlegrean mandarin analyzed in different components (peel, pulp, and seed) at different ripening stages of the fruit (unripe fruits—UF, semi-ripe fruits—SRF, ripe fruits—RF). The tree of the heatmap has two main clusters. The first (I) includes two branches that separate the pulp from the seed (UF and SRF) phytochemicals. In addition, the second cluster (II) includes two branches characterized by the highest value of antioxidant activity: all compounds analyzed in the peel which are divided from compounds analyzed in the seed, RF. The pulp is the component with the lowest quantity of found phytochemicals. The seed exhibits greater antioxidant capacity, DPPH radical scavenging activity and ascorbic acid content with the highest values at the ripe stage. The peel contains higher levels of polyphenols, chlorophylls, carotenoids, and flavonoids. Polyphenols and chlorophylls increase in the peel at the unripe stage, whereas carotenoids are higher during the ripe stage. Both seed and peel show more tannins during the ripe stage compared to the pulp.

### 3.7. Interaction between Fruit Component and Ripening Stages

Data shown in Table 3 indicated that the content of phytochemicals in Phlegrean mandarin was affected by both different components of fruit (C) and fruit ripening stages (RS), as well as by their interaction (C × RS). Among fruit components, peel was the richest in total antioxidant capacity (TAC), total polyphenol content (TP), total flavonoids (TF), total chlorophylls (TCHL) and carotenoids (TCAR), while seeds showed the highest value for DPPH radical scavenging activity, total condensed tannins (TCT) and ascorbic acid (AsA). The ripening stage with the highest antioxidant content was the final stage when fruits reached full maturation and were ready to be harvested and consumed.

The combination Seed × RF was the best for TAC, DPPH, TCT and AsA content, while Peel × UF showed the highest TP and TS amounts. These data confirm the by-products of Phlegrean mandarin to be a valuable source of antioxidants, more than largely consumed pulp.

## 4. Discussion

In this work, we explore the possibility that waste products of Phlegrean mandarin, namely seeds and peel, may be recycled as a primary source of bioactive compounds beneficial for human health. Starting from the evidence that the by-products of this specific cultivar may represent a mine of antioxidants [23], we have further deepened our study, investigating if and how the ripening process may affect the bioactive compound modulation in different fruit parts. In the last few decades, there was an increasing interest in searching for natural molecules which may be used as food supplements to contrast several diseases [37]. Fruits contain many antioxidant compounds in a different mixture, depending on several factors, including component, cultivar, species, and maturation stage [7]. Our results evidenced that phenols, flavonoids, condensed tannins, and ascorbic acid contribute to the high antioxidant activity found in peel and seeds of the Phlegrean mandarin. Moreover, ripening influences the amount of these bioactive compounds, which was particularly abundant in the final stage of fruit maturation as regards tannins, flavonoids, and ascorbic acid. Our data are consistent with previous findings on other citrus cultivars where the progression of the ripening modulates the concentrations of phenols, anthocyanins, and carotenoids that increase with maturation conferring the final organoleptic characteristics and distinct flavour to fruits [38,39].

Even if our data demonstrated that all parts of the fruit are an excellent source of antioxidant compounds, the most substantial concentration measured in peel and seeds, generally considered by-products, opens a new scenario for innovative uses of the waste components of citrus, mainly in food and pharmaceutical industries [23]. In a recent study, Caggia et al. [40] demonstrated that the addition of debittered orange fiber was compatible with bakery products, replacing the fat component, and improving nutritional traits of a bakery confectionary products, leading to a low-fat brioche fortified with natural fiber being obtained.

Phenolic compounds represent a significant group of water-soluble antioxidants, including three classes: simple phenols, flavonoids, and tannins. They are widespread in almost all plant tissues. At the physiological level, they act as scavengers of ROS produced by the photosynthetic electron transport; from a functional point of view, phenolic compounds are involved in the resistance to pathogens and defense against predators by conferring tissues and fruits the typical astringent taste, which negatively affect the palatability [41].

Consistent with findings of other authors [42,43,44,45,46], our data demonstrated that these compounds are not uniformly distributed within the fruit tissues, being mainly located in the epidermis and sub-epidermis layers of the fruit.

The high concentration of polyphenols in the peel is likely associated with their active role in photoprotection against UV radiation and in the attraction of pollinators to allow the dispersion of seeds and defense mechanisms against pathogens and predators [47].

A comparative study evidenced that Phlegrean mandarin (*C. reticulata* Blanco) exhibited the highest concentration of total polyphenols in peel and seeds than the most commercial varieties *C. clementina* and *C. japonica* [23].

In our study, the concentrations of total polyphenols ranged from 0.80 (in pulp and seeds of UF and SRF) to 5.17 (peel and mature seeds) mg GAE g^−1^ FW, according to values reported in Moulehi et al. [7].

Flavonol glycosides, belonging to the phenolic compounds, are present in almost all plants and strongly influence the fruit color. They are mainly located in the peel of fruits since light is required for their synthesis. Our data showed that in *C. reticulata*, peel was rich in flavonoids.

It is noteworthy that climatic factors (i.e., temperature and irradiance) affect these metabolites in plants [48]. Thus, conditions of full sunlight (characteristic of the Phlegrean area), could have induced the accumulation of flavonoids in the peel of this specific cultivar [49].

Another added value of the Phlegrean mandarin cultivar is the high concentration of tannins in the by-products of peel and seeds, especially in mature fruits [23]. As previously observed in many plant species, the presence of elevated levels of tannins in this cultivar is linked to plant capability to cope with pathogens and herbivores [50,51].

As reported in several studies, the tannins content and, in general, phenolic compounds, should decrease with ripening. This trend has an evolutionistic meaning: plants need to spread the seed for the propagation of the species, and in this regard, astringency decreases with the ripening of fruits to make them more appetizing [52]. Vázquez-Gutiérrez et al. [53] found that the tannin content of fresh samples decreased significantly as ripening progressed.

In Phlegrean mandarin, only in the peel, we observed a decrease of total polyphenols with ripening, while the opposite was observed for tannins and flavonoids, which increased with fruit maturation. The ripening did not affect the phenolic compounds in the pulp.

A peculiar behavior was found in seeds where the content of total polyphenols, flavonoids and condensed tannins increased as ripening progressed, showing the highest values at the mature stage. We suppose that the modulation of these compounds with ripening is due to intrinsic characteristics of the species [41], considering that the phenol content is strongly affected by many factors, i.e., cultivar, species, ripeness, harvest time, climatic conditions, storage time and environment [54,55,56].

The high presence of phenolic compounds in Phlegrean mandarin seeds focuses on the possibility of utilizing seeds as a source of food supplement.

The peculiar results found in peel and even more in seeds encouraged us to identify the specific profiles of phenolic compounds present in peel and seeds at different ripening stages.

The comparison of values reported in Table 1 and Table 2 evidenced the presence of high amounts of colourful anthocyanins in the peels, with delphinidin derivatives as the main products; in seeds, the hydrolysable tannin valoneic acid dilactone was the main product.

These data suggest that the antioxidant properties found in peel are due mainly to anthocyanins. On the other hand, the free-radical scavenging and antioxidant activity of anthocyanin pigments are well documented and justify their roles as medicinal agents in folk medicine throughout the world, since these pigments are linked to a wide range of health benefits [57,58,59]. It is significant that just recently delphinidin-3-*O*-glucoside has been recognized as a potential anti-inflammatory of TNF-α signaling, an inflammatory cytokine that is overexpressed in metabolic syndrome [60].

Among detected flavonoids, rutin is a citrus glycoside with neuroprotective properties for the treatment of neurodegenerative diseases (NDs), naringin is involved in anti-inflammatory, anti-cancer activities, bone regeneration, metabolic syndrome, oxidative stress, genetic damage, and central nervous system (CNS) diseases [61], and quercetin is in ongoing studies regarding COVID-19 [62].

In seeds, the antioxidant activity is mainly due to tannins and polyphenols. In particular, valoneic acid dilactone has a significant antidiabetic activity [63]. As in peel, seeds of Phlegrean mandarin also contain cyanidin-3-*O*-glucoside, delphinidin-3-*O*-glucoside, naringin, gallic acid, rutin and quercetin-3-glucoside in concentrations higher than those found in seeds of other citrus cultivars [7], enhancing the nutraceutical value of Phlegrean mandarin.

Concerning ripening stages, our results showed that, in peel, the highest concentration of phenolic compounds were found in SMF, while in seeds, appreciable levels of these molecules were detected in UF.

Peel and seeds of Phlregrean mandarin are also a source of ascorbic acid. Vitamin C is one of the main compounds in citrus fruit and an essential dietary nutrient to prevent scurvy, cancer, cardiovascular and chronic nervous system diseases [64]. Many factors influence vitamin C content, namely species and cultivar and rootstock, climate, maturity stage, fruit position on the tree, harvest, and storage conditions [41]. According to Fattahi et al. [65], our results showed that the concentration of ascorbic acid increased during ripening in peel and seeds, while it decreased in the pulp. It has been proposed that the rise of ascorbic acid during ripening may be a way to balance the decrease of phenolic compounds as maturation progresses, preserving fruits from oxidative damages [66].

Data collected for pigments (chlorophyll and carotenoids) confirm that the most valuable fruit components are the by-products [7].

In agreement with Agócs et al. [67], the total pigment content was very high in the peel compared to the pulp and seeds in all ripening stages. The unripe mandarins contained the higher chlorophyll concentration in both peel and seeds. However, according to other authors [67,68,69], ripening resulted in a significant decrease of chlorophyll, especially in the peel, due to the degradation of these pigments replaced by the synthesis of carotenoids, i.e., β-cryptoxanthin and 9-cis-violaxanthin [26,70]. It has been demonstrated that the drop of daily temperature during the cold season when mandarin fruits are harvested activates the chlorophyllase enzyme, resulting in the degradation of chlorophyll and the appearance of carotenoids.

In seeds, the decrease of chlorophyll content could be due to the involvement of chlorophyll in activating light-harvest mechanisms to promote germination [71]. Although not much information is available on the functions of carotenoids in seeds; previous research has shown that carotenoids would exert a key role in the production of abscisic acid (ABA) and in the induction of seed dormancy [72], one of the mechanisms by which plants can delay germination when environmental conditions are unfavorable [73]. Therefore, the lower concentration of carotenoids in *C. reticulata* seeds compared to peel and pulp in all ripening stages was likely due to the distribution of this cultivar in Mediterranean ecosystems where the mild climate favors germination [23].

As antioxidants, carotenoids counteract the deterioration of membranes induced by free radicals and ageing [74], contributing to the improvement of the fruit quality [75].

In our paper, the antioxidant activity of the peel, pulp, and seeds extracts was evaluated by two complementary methods, namely FRAP and DPPH assays, to validate the results. Our data confirmed that ripening strongly influences the antioxidant activities, as previously demonstrated by Fattahi et al. [65] and Moulhehi et al. [7]. Several studies [41,76] report a significant decrease in antioxidant activity with maturation. This trend was confirmed only in the peel of Phlegrean mandarin. A drop of antioxidant activity with ripening could be due to an irregular deposition of phenolic compounds in tissues [76] or reduced antioxidant enzymes such as ascorbate and glutathione. Racchi [41] reported that the decline of the antioxidant system in sweet orange fruits occurred simultaneously with the reduction of antioxidant activity due to the increase of oxidative stress with maturation. In these circumstances, the ripening and senescence processes, resulting in ROS accumulation, may have affected the antioxidant activity.

A particularly interesting result is that, in Phlegrean mandarin, the seed antioxidant activity was enhanced by ripening, according to other studies [70,77,78].

## 5. Conclusions

Phlegrean mandarin is confirmed as an excellent source of bioactive compounds, whose concentration changes according to the fruit component and the ripening stage. The clustering tree of the heatmap identifies the ‘component’ as the main discriminant factor compared to the ‘ripening stage’. In particular, seeds showed the highest antioxidant activity, confirming our previous results [23], and peel was the fruit component richest in phenolic compounds.

Our data indicates that the by-product of mandarin are valuable sources of antioxidants and suggest a potential use of citrus wastes for innovative applications (phytochemicals or additives for functional foods) by selecting the component and ripening stage with the highest phytochemical content.

Phytochemicals, such as flavonoids, namely naringin, hesperidin, and rutin, are actively used in pharmaceutical applications. Moreover, recently, quercetin has been recommended as a potential molecule candidate for an anti-COVID-19 role. Furthermore, the significant changes of the phenolic compositions during ripening suggest that a collection of these parts at appropriate stages of fruit maturation must be considered to maximize the occurrence of determined bioactive compounds and their possible related utilization. In addition, the recycling of such components would reduce the environmental impact due to the vast quantity of wastes derived annually from the agri-food industry. However, further biological and pharmacological studies are needed to demonstrate and elucidate the health benefits of citrus wastes for human nutrition.

## Figures and Tables

**Table 1:** Variation of phenolic composition in peel of Phlegrean mandarin during fruit ripening (unripe fruits—UF, semi-ripe fruits—SRF, ripe fruits—RF). Values are means of five replicates ± SD.

Compounds	UF (µg L^−1^)	SRF (µg L^−1^)	RF (µg L^−1^)
Delphinidin-3,5-diglucoside	3.07 ± 0.20	4.14 ± 0.22	1.84 ± 0.09
Cyanidin-3,5-di-*O*-glucoside	2.87 ± 0.11	2.26 ± 0.14	3.16 ± 0.14
Delphinidin-3-*O*-glucoside	2032.45 ± 177.38	2644.42 ± 96.01	2102.92 ± 115.58
Cyanidin-3-*O*-glucoside	154.87 ± 8.48	161.96 ± 11.32	88.26 ± 3.04
Delphinidin-3-*O*-arabinoside	8.55 ± 0.30	6.21 ± 0.61	2.49 ± 0.10
Petunidin-3-*O*-glucoside	60.47 ± 4.32	53.93 ± 4.76	53.80 ± 2.95
Cyanidin-3-*O*-arabinoside	1.92 ± 0.14	1.15 ± 0.07	-
Pelargonidin-3-*O*-glucoside	75.07 ± 3.21	53.23 ± 1.83	27.84 ± 1.14
Peonidin-3-*O*-glucoside	143.74 ± 19.28	199.68 ± 10.57	339.94 ± 6.79
Malvidin-3-O-glucoside	82.20 ± 3.65	70.21 ± 5.07	86.93 ± 4.52
Malvidin-3-*O*-arabinoside	18.34 ± 1.19	22.43 ± 0.79	9.79 ± 0.43
Delphinidin rutinoside	4019.47 ± 304.32	4407.85 ± 266.85	3255.32 ± 219.63
Malvidin 3-*O*-p-coumaroylglucoside	16.96 ± 0.80	11.59 ± 0.44	17.41 ± 0.36
Naringin	391.61 ± 20.89	445.15 ± 26.70	40.56 ± 3.12
Quercetin-3-glucoside	2083.89 ± 104.38	2654.77 ± 144.51	2048.06 ± 119.97
EGC 3-gallate	-	6.26 ± 0.52	10.33 ± 0.46
GC 3-gallate	-	4.53 ± 0.50	9.50 ± 0.71
Gallic acid	52.09 ± 2.09	42.42 ± 2.21	47.66 ± 2.42
Syringaldehyde	3.30 ± 0.12	5.19 ± 0.45	9.69 ± 0.43
Syringic acid	32.83 ± 1.14	20.91 ± 1.23	41.64 ± 2.48
Chlorogenic acid	68.90 ± 5.43	81.74 ± 4.25	53.68 ± 1.45
Quercetin-3-*O*-rhamnoside	76.12 ± 4.04	76.99 ± 1.15	56.09 ± 4.65
Valoneic acid dilactone	1430.20 ± 68.58	103.41 ± 12.63	268.53 ± 10.98
Phloretin	9.77 ± 0.60	8.66 ± 0.58	9.68 ± 0.83
Phloridzin	19.75 ± 0.74	20.90 ± 2.47	19.51 ± 0.80
Myricitrin	137.26 ± 11.79	83.68 ± 8.06	83.54 ± 1.89
Sinensetin	4164.48 ± 265.24	2024.25 ± 228.68	1769.17 ± 117.64
Rutin	3608.01 ± 175.96	4055.79 ± 207.04	3610.47 ± 222.90

**Table 2:** Variation of phenolic composition in seeds of Phlegrean mandarin during fruit ripening (unripe fruits—UF, semi-ripe fruits—SRF, ripe fruits—RF). Values are means of five replicates ± SD.

Compounds	UF (µg L^−1^)	SRF (µg L^−1^)	RF (µg L^−1^)
Delphinidin diglucoside	0.36 ± 0.02	-	-
Cyanidin-3,5-di-*O*-glucoside	14.07 ± 0.86	35.19 ± 1.85	22.75 ± 0.92
Delphinidin-3-*O*-glucoside	66.61 ± 7.17	19.76 ± 2.06	11.27 ± 0.76
Cyanidin-3-*O*-glucoside	174.57 ± 6.46	293.95 ± 8.49	345.78 ± 22.55
Delphinidin-3-*O*-arabinoside	15.38 ± 1.42	5.25 ± 0.26	9.69 ± 0.35
Petunidin-3-*O*-glucoside	40.81 ± 6.08	16.91 ± 0.78	24.25 ± 2.92
Cyanidin-3-*O*-arabinoside	7.07 ± 0.47	9.31 ± 0.50	23.97 ± 1.65
Pelargonidin-3-*O*-glucoside	54.79 ± 4.01	35.73 ± 3.11	40.58 ± 1.41
Peonidin-3-*O*-glucoside	34.72 ± 2.20	-	14.09 ± 1.05
Malvidin-3-*O*-glucoside	2.15 ± 0.35	-	4.64 ± 0.50
Malvidin-3-*O*-arabinoside	3.21 ± 0.12	-	-
Delphinidin rutinoside	56.16 ± 3.33	16.14 ± 1.24	3.83 ± 0.11
Malvidin 3-*O*-p-coumaroylglucoside	2.51 ± 0.09	-	-
Naringin	15.31 ± 1.07	3.03 ± 0.13	4.31 ± 0.20
Quercetin-3-glucoside	40.21 ± 2.20	15.56 ± 1.11	6.14 ± 0.35
Procyanidin B1	6.61 ± 0.90	-	-
Catechin	0.67 ± 0.07	11.09 ± 0.79	8.74 ± 0.69
Epicatechin	1.86 ± 0.16	1.59 ± 0.10	7.22 ± 0.25
Catechin-3-gallate	12.35 ± 0.57	18.46 ± 0.98	14.93 ± 0.73
EC-3-gallate	12.40 ± 1.58	16.59 ± 0.83	12.44 ± 1.46
EGC 3-gallate	21.74 ± 1.18	9.08 ± 0.94	8.03 ± 0.33
GC 3-gallate	21.29 ± 0.71	18.10 ± 0.66	9.26 ± 0.55
Gallic acid	71.80 ± 5.73	66.27 ± 3.43	28.93 ± 1.46
Syringaldehyde	6.82 ± 0.42	0.69 ± 0.02	-
Syringic acid	40.33 ± 2.63	18.85 ± 0.63	12.11 ± 1.28
6-Malonyldaidzin	109.00 ± 5.60	67.11 ± 4.24	143.32 ± 14.93
Chlorogenic acid	44.56 ± 4.52	32.18 ± 1.31	36.30 ± 1.71
Quercetin-3-*O*-rhamnoside	8.81 ± 0.50	-	-
Valoneic acid dilactone	13.127.81 ± 744.41	5947.83 ± 461.67	11.649.89 ± 559.83
Phloretin	39.84 ± 2.32	9.31 ± 0.43	10.38 ± 1.10
Phloridzin	9.21 ± 0.60	4.34 ± 0.19	-
Myricitrin	74.35 ± 3.26	25.49 ± 1.87	41.91 ± 2.90
Sinensetin	7.38 ± 0.60	-	-
Rutin	54.54 ± 5.28	17.61 ± 1.43	3.80 ± 0.19

**Table 3:** Analysis of variance and means comparison for bioactive compounds in Phlegrean mandarin in response to different components (C) (Peel, Pulp, Seed) and ripening stage (RS) (unripe fruits—UF, semi-ripe fruits—SRF, ripe fruits—RF), as well as nine different combinations C × RS. Different letters within each column indicate significant differences according to Duncan’s multiple comparison tests (*p* < 0.05). Asterisks (*) represent the level of significance for main factors (C, RS) and their interaction (C × RS): NS—not significant; *** *p* < 0.001.

	TAC	DPPH	TP	TF	TCT	AsA	TCHL	TCAR
C								
Peel	17 a	42	4.2 a	17 a	10 b	4.3 b	0.23 a	0.15 a
Pulp	4.8 b	14	0.9 c	0.6 c	4.0 c	3.6 b	0.05 c	0.04 b
Seed	17 a	62	2.1 b	6.3 b	11 a	12 a	0.12 b	0.02 c
**RS**								
UF	10 c	36	2.3 b	8.0 a	7.6 b	4.9 c	0.24 a	0.04 c
SRF	13 b	38	1.9 c	6.9 b	6.5 c	6.9 b	0.06 b	0.07 b
RF	16 a	43	3.0 a	8.6 a	12 a	8.4 a	0.09 b	0.11 a
**Interaction**								
Peel × UF	19 c	47 d	5.2 a	18 a	10 b	1.6 f	0.50 a	0.06 c
Pulp × UF	5.7 f	9.8 h	0.9 e	0.5 e	3.7 d	4.2 e	0.06 c	0.02 d
Seed × UF	5.8 f	51 c	0.8 e	5.7 d	8.9 b	8.7 c	0.17 b	0.03 d
Peel × SRF	16 e	42 e	3.9 c	15 b	5.7 c	4.2 e	0.10 b	0.14 b
Pulp × SRF	3.7 g	11 h	0.8 e	0.5 e	3.8 d	4.3 e	0.03 c	0.05 c
Seed × SRF	20 b	63 b	1.0 e	5.1 d	10 b	12 b	0.06 c	0.01 d
Peel × RF	17 d	37 f	3.5 d	17 a	15 a	6.9 d	0.10 b	0.24 a
Pulp × RF	5.1 f	21 g	0.9 e	0.7 e	4.6 d	2.2 f	0.04 c	0.06 c
Seed × RF	24 a	72 a	4.4 b	8.0 c	15 a	16 a	0.12 b	0.02 d
**Significance**								
C	***	***	***	***	***	***	***	***
RS	***	***	***	***	***	***	***	***
C × RS	***	***	***	***	***	***	***	***

TAC: total antioxidant capacity (μmol TE g^−1^ FW); DPPH: DPPH radical scavenging activity (% inhibition); TPC: total polyphenol content (mg GAE g^−1^ FW); TF: total flavonoids (mg CE g^−1^ FW); TCT: total condensed tannins (mg CE g^−1^ FW); AsA: ascorbic acid (ng μL^−1^); TCHL: total chlorophylls (mg g^−1^ FW); TCAR: total carotenoids (mg g^−1^ FW).

## Data Availability

The data presented in this study are available in this article.

## Supplementary Materials

The following supporting information can be downloaded at: https://www.mdpi.com/article/10.3390/antiox11020187/s1, Table S1: Mass Spectra parameters: list of precursor ion, product ions, declustering potential (DP) and collision energy (CE).
