# Supplementary material for: Antioxidant Properties of Pulp, Peel and Seeds of Phlegrean Mandarin (Citrus reticulata Blanco) at Different Stages of Fruit Ripening
Source: Antioxidants (Basel). 2022 Jan 19;11(2):187. doi: 10.3390/antiox11020187 (PMC8868052; doi:10.3390/antiox11020187)
Supplement: Supplementary file 1 [file antioxidants-11-00187-s001.zip › antioxidants-1553146-supplementary.pdf]

Supplementary Table S1. Mass Spectra parameters: list of precursor ion, product ions, declustering potential (DP) and collision energy (CE).

Compound	Precursor ion	Product ion	DP	CE
Delphinidin diglucoside	627	303	90	46
Delphinidin diglucoside	627	256	90	46
Cyanidin-3,5-di-*O*-glucoside	611	287	177	55
Delphinidin-3-*O*-glucoside	465	303	181	30
Delphinidin-3-*O*-glucoside	465	285	181	30
Cyanidin-3-*O*-glucoside	449	287	192	27
Cyanidin-3-*O*-glucoside	449	241	192	27
Delphinidin-3-*O*-arabinoside	435	303	192	25
Delphinidin-3-*O*-arabinoside	435	285	192	25
Petunidin-3-*O*-glucoside	479	317	172	27
Petunidin-3-*O*-glucoside	479	274	172	27
Cyanidin-3-*O*-arabinoside	419	287	194	24
Cyanidin-3-*O*-arabinoside	419	241	194	24
Pelargonidin-3-*O*-glucoside	433	271	163	28
Petunidin-3-*O*-arabinoside	449	317	124	25
Petunidin-3-*O*-arabinoside	449	274	124	25
Peonidin-3-*O*-glucoside	463	301	104	29
Peonidin-3-*O*-glucoside	463	268	104	29
Malvidin-3-*O*-glucoside	493	331	94	29
Malvidin-3-*O*-glucoside	493	270	94	29
Malvidin-3-*O*-arabinoside	463	331	94	24
Malvidin-3-*O*-arabinoside	463	270	94	24
Delphinidin rutinoside	611	303	165	45
Delphinidin rutinoside	611	284	165	45
Malvidin 3-*O*-p-coumaroylglucoside	639	331	121	29
Malvidin 3-*O*-p-coumaroylglucoside	639	315	121	29
Naringin	581	273	105	50
Naringin	581	153	105	50
Flavone+Na	245	227	98	32
Flavone+Na	245	209	98	32
Apigenin	271	153	171	49
Apigenin	271	271	171	49
Quercetin-3-glucoside	465	303	104	29
Quercetin-3-glucoside	465	245	104	29
Procyanidin B1	579	291	105	50
Procyanidin B1	579	409	105	50
Catechin	291	139	16	21
Catechin	291	123	16	21
Epicatechin	291	138	36	21

Epicatechin	291	123	36	21
Caffeine	195	138	71	26
Caffeine	195	110	71	26
Catechin-3-gallate	443	291	124	25
Catechin-3-gallate	443	273	124	25
EC-3-gallate	443	291	124	25
EC-3-gallate	443	273	124	25
EGC 3-gallate	459	307	104	29
EGC 3-gallate	459	289	104	29
Gallocatechin	307	291	81	20
Gallocatechin	307	139	81	20
GC 3-gallate	459	307	104	29
GC 3-gallate	459	289	104	29
Gallic acid	171	127	59	20
Gallic acid	171	109	59	20
Syringaldehyde	183	123	41	17
Syringaldehyde	183	77	41	17
Syringic acid	199	140	16	21
Syringic acid	199	155	16	21
6-Malonyldaidzin	503	485	104	29
6-Malonyldaidzin	503	467	104	29
Daidzein	255	237	102	28
Daidzein	255	227	102	28
Eriodictyol	289	247	88	22
Eriodictyol	289	271	88	22
Quercetin	303	285	30	32
Quercetin	303	276	30	32
Quercetin	303	258	30	32
Chlorogenic acid	355	163	50	15
Chlorogenic acid	355	145	50	15
Quercetin-3-*O*-rhamnoside	449	303	104	29
Quercetin-3-*O*-rhamnoside	449	285	104	29
Valoneic acid dilactone	471	453	104	29
Valoneic acid dilactone	471	425	104	29
Phloretin	275	169	20	25
Phloretin	275	125	20	25
Phloridzin	437	275	97	15
Phloridzin	437	169	97	15
Myricetin	320	153	80	35
Myricetin	320	181	80	35
Myricitrin	465	465	97	25
Myricitrin	465	318	97	25
Kaempferol	287	153	120	28

Kaempferol	287	219	120	28
Sinensetin	373	343	80	10
Sinensetin	373	321	80	10
Rutin	611	303	104	30
Rutin	611	272	104	30
Procyanidin C	867	579	105	50
Procyanidin C	867	291	105	50
Procyanidin tetramer	1155	867	105	50
Procyanidin tetramer	1155	579	105	50
